# The association of month of birth with allergic sensitization in pediatric patients with asthma in Mureş County, Romania

**DOI:** 10.1186/2045-7022-3-S3-P165

**Published:** 2013-07-25

**Authors:** B Capilna, C Radu

**Affiliations:** 1 Pediatric Department, University of Medicine and Pharmacy Targu Mures, Targu Mures, Romania; 2 Pediatric Clinic; 3 Department of Cardiovascular Surgery, County Emergency Hospital Targu Mures, Targu Mures, Romania

## Background

It is well known that allergic diseases occur as a consequence of genetic and environmental interactions.Early infancy seems to be a period of particular susceptibility to sensitization, as indicated by epidemiological and experimental studies.

## Methods

Our prospective study included eighty-eight asthmatic children, with ages between 1 and 18 years, who were admitted to the Clinic of Pediatrics I from Târgu Mureş, Romania, between October 2008 and June 2010. We analyzed the age, month of birth, sensitization to a given antigen (Dermatophagoides pteronyssinus, Dermatophagoides farinae, milk proteins, egg, mold, dog epithelium, cat epithelium, soya, carrot, potatoes, peanuts, tomato). Specific Ig E serum levels to allergens were measured. RAST equal or higher than class 1 was considered as positive. This data was combined with the presence of different types of allergens during the year.

## Results

The link between the month of birth and the incidence of allergic disease was statistically significant. We observed the appearance of three “waves”, in February, April, September and early October the incidence of allergy being higher (between 13.2% and 15.5%) than in the other months (under 9.09%, with the lowest value of 1.9% in November. Type class 6 allergic sensitization is more common in July, type 5 in September, type 4 and 3 in April, and type 2 and 1 in February.

## Conclusion

Our results support the hypothesis that the first few months of life represent a sensitive period, during which protection from exposure to pollen allergens may be associated with decreased sensitization to pollens.

## Disclosure of interest

None declared.

